# The Colony-Stimulating Factor-1 (CSF-1) Receptor Sustains ERK1/2 Activation and Proliferation in Breast Cancer Cell Lines

**DOI:** 10.1371/journal.pone.0027450

**Published:** 2011-11-09

**Authors:** Andrea Morandi, Valentina Barbetti, Maria Riverso, Persio Dello Sbarba, Elisabetta Rovida

**Affiliations:** Dipartimento di Patologia e Oncologia Sperimentali, Università degli Studi di Firenze, and Istituto Toscano Tumori, Firenze, Italy; University of Chicago, United States of America

## Abstract

Breast cancer is the second leading cause of cancer-related deaths in western countries. Colony-Stimulating Factor-1 (CSF-1) and its receptor (CSF-1R) regulate macrophage and osteoclast production, trophoblast implantation and mammary gland development. The expression of CSF-1R and/or CSF-1 strongly correlates with poor prognosis in several human epithelial tumors, including breast carcinomas. We demonstrate that CSF-1 and CSF-1R are expressed, although at different levels, in 16/17 breast cancer cell lines tested with no differences among molecular subtypes. The role of CSF-1/CSF-1R in the proliferation of breast cancer cells was then studied in MDAMB468 and SKBR3 cells belonging to different subtypes. CSF-1 administration induced ERK1/2 phosphorylation and enhanced cell proliferation in both cell lines. Furthermore, the inhibition of CSF-1/CSF-1R signaling, by CSF-1R siRNA or imatinib treatment, impaired CSF-1 induced ERK1/2 activation and cell proliferation. We also demonstrate that c-Jun, cyclin D1 and c-Myc, known for their involvement in cell proliferation, are downstream CSF-1R in breast cancer cells. The presence of a proliferative CSF-1/CSF-1R autocrine loop involving ERK1/2 was also found. The wide expression of the CSF-1/CSF-1R pair across breast cancer cell subtypes supports CSF-1/CSF-1R targeting in breast cancer therapy.

## Introduction

The c-*fms* proto-oncogene encodes the only known receptor (CSF-1R) for Colony Stimulating Factor 1 (CSF-1 or M-CSF) [1], [2]. CSF-1R is a class III transmembrane tyrosine kinase receptor and its ligand CSF-1 has secreted glycoprotein, secreted proteoglycan and membrane-bound isoforms [3], [4]. The CSF-1/CSF-1R pair has essential physiological functions in the generation of osteoclasts and macrophages [4] and, via its action on macrophages and other CSF-1R-expressing cells, in female and male fertility [5], [6]. Activation of CSF-1R by its ligand triggers a series of rapid events, including receptor dimerization and tyrosine phosphorylation of its intracellular domain. Phosphorylation at particular CSF-1R tyrosines creates binding sites for a variety of cytoplasmic proteins that activate signal transduction pathways including that of ERK1/2 and PI3K [7].

CSF-1 and CSF-1R are expressed in normal breast tissue during puberty, pregnancy and lactation. However, the expression of CSF-1R and/or CSF-1 has been documented in several human cancers, including carcinomas of breast, female reproductive tract, prostate and kidney [8]–[15]. Data reported in literature for solid tumors indicate that the oncogenic potential of CSF-1/CSF-1R is due to the co-expression of this growth factor/receptor pair, rather than CSF-1R overexpression or mutations activating CSF-1R independently of ligand [6]. This is supported by the fact that the expression of normal c-*fms* into CSF-1-expressing non-transformed fibroblasts and epithelial cells can be sufficient to induce a fully transformed phenotype [16], [17]. In this respect, activation of CSF-1R by its ligand is likely to occur in tumor cells in which CSF-1R and CSF-1 are co-expressed (i.e. autocrine activation), or when CSF-1R is stimulated by CSF-1 released by cancer associated fibroblasts (i.e. paracrine activation). Consistent with this, in breast cancer patients, the expression of both CSF-1 and its receptor in neoplastic epithelial cells strongly correlates with poor prognosis and is predictive of ipsilateral recurrence [18]–[20]. In addition, the presence of tumor associated macrophages in breast tumors also correlates with poor prognosis [19], [21] and, in mouse models, CSF-1 promotes metastasis [22], stimulates angiogenesis [23], [24] and is involved in a paracrine loop with EGF to promote tumor cell invasion [25]. While previous studies indicated that CSF-1R and CSF-1 are expressed in breast cancer cell lines and tumors and demonstrated the relevance of CSF-1/CSF-1R signaling in the invasiveness of breast cancer cells [26]–[31], few studies have focused on the biological role of CSF-1/CSF-1R signaling in the proliferation of breast cancer cells.

Targeting receptor tyrosine kinases with kinase inhibitors (e.g. imatinib, dasatinib or nilotinib) has recently opened a new era in the treatment of hematologic malignancies and solid tumors such as gastrointestinal stromal tumors [32], [33]. These drugs are effective on CSF-1R [34], [35] and other CSF-1R-specific inhibitors have been developed [36]–[38]. More importantly, several drugs targeting CSF-1 and CSF-1R are currently in Phase I/II trial (www.clinicaltrials.org). Elucidation of the involvement of CSF-1R in breast cancer cell proliferation would strengthen the rationale of CSF-1R targeting in CSF-1R expressing cancers.

In this work, we characterized the role of CSF-1R in the proliferation of breast cancer cells and found that CSF-1R is widely expressed in breast cancer cell lines at both mRNA and protein levels. Interfering with the CSF-1/CSF-1R signaling pathway, either by CSF-1R inhibition or by inhibition of autocrine CSF-1, impaired MDAMB468 and SKBR3 cell proliferation. In addition, exposure to ectopic CSF-1 stimulated MDAMB468 and SKBR3 growth. We found ERK1/2, c-Jun, cyclin D1 and c-Myc, known for their involvement in cell proliferation, to be downstream CSF-1R in breast cancer cells. The wide expression of CSF-1/CSF-1R pair across breast cancer cell subtypes supports CSF-1/CSF-1R targeting in breast cancer therapy.

## Materials and Methods

### Cells and cell culture

NIH/3T3 murine fibroblasts expressing ectopic human CSF-1R (kind gift of MF Roussel, St. Jude Children's Research Hospital, Memphis, TN, USA) [39] and HepG2 human hepatoblastoma cells (www.lgcstandards-atcc.org) periodically tested in our laboratory by western blotting for the presence of EGFR protein) were cultured in DMEM, while human chronic myeloid leukemia K562 cells (www.lgcstandards-atcc.org); periodically tested in our laboratory by western blotting or Q-PCR for the expression of BCR/Abl) in RPMI, supplemented with 4 mM glutamine and 10% fetal bovine serum (FBS). Human primary macrophages were obtained after informed consent as previously described [40]. The breast cancer cell lines (MCF7, T47D, MDAMB175VII, ZR751, 734B, MDAMB361, BT474, SKBR3, MDAMB453, HCC1954, MDAMB468, BT20, SUM149PT, HCC1500, MDAMB231) and MCF10A and MCF12A cells (two immortal, non transformed cell lines showing basal B molecular pattern) [41], [42] were a kind gift of Dr MG Daidone and Dr E Tagliabue, Istituto Nazionale Tumori, Milano, Italy; Dr D Lerouge, Institut Gustave-Roussy, Villejuif, France; Prof CM Isacke, Institute of Cancer Research, London, UK. Laboratories of origin have tested all cell lines by microsatellite analysis or microarray. However, cells have been tested upon arrival and periodically in our lab by western blotting for HER2, EGFR and by PCR for estrogen receptor expression. Cells were cultured as previously described [41]. Cells were incubated in the presence or the absence of 100 mg/ml streptomycin and 100 I.U. penicillin, at 37°C in humidified atmosphere containing 5% CO_2_.

### Analysis of Gene Expression and cGH Datasets

Data were obtained mapping CSF-1 and CSF-1R gene symbols to GSE2603 (probes 2078082_at and 203104_at) [43], NKI (probes NM_000757 and NM_000971) [44] and Neve (probes 2078082_at and 203104_at) [41] datasets. Cell lines molecular subtypes were reported as classified by the authors in the Neve dataset [41]. Breast cancer subtypes of tumor samples were predicted using centroid Spearman correlation to the PAM50 classifier in NKI and GSE2603 datasets. The PAM50 gene expression predictor classifies breast cancers into molecular intrinsic subtypes (Luminal A, Luminal B, HER2-enriched, Basal-like) and provides a risk of recurrence (ROR) score based on the similarity of an individual sample to prototypic subtypes [45]. GSE2603 data were GCRMA normalized, NKI data were mean centered normalized and the Neve data were RMA normalized as reported by the authors in their studies using the ROCK database [46]. The CGH array data for CSF-1R and CSF-1 were obtained from Neve [41] and Fridlyand [47] (RP1-141L3 and CTD-2050A15). Statistical analysis was performed using Prism software (GraphPad Software, La Jolla, CA, USA). Means of the normalized data of each subtype were compared using one-way ANOVA and the Bonferroni test. Differences were considered statistically significant when p<0.05. We did not submit our research to the local ethical committee nor did we obtain informed consent for the use of tumor explants because our analyses were performed on publicly available datasets (see below).

### Total cell lysates

Culture plates were placed on ice, cell monolayers rapidly washed 3 times with ice-cold PBS containing 100 mM orthovanadate and cells lysed by scraping in Laemmli buffer (Tris/HCl 62.5 mM, pH 6.8, 10% glycerol, 0.005% blue bromophenol, 2% SDS) and incubating at 95°C for 10 minutes in the presence of 100 mM 2-mercaptoethanol. Lysates were then clarified by centrifugation (20000 g, 10 minutes, RT).

### Western Blotting and Immunoblotting

30–60 µg of total proteins was separated by SDS-PAGE in 9–15% polyacrylamide gel and transferred onto PVDF membranes (Millipore) by electroblotting. Membranes were incubated (1 hour, RT) in Odyssey Blocking Buffer diluted 1∶1 with PBS, and then in the same buffer containing 0.1% Tween-20 and the primary antibody (16–18 hours, 4°C). After extensive washing with PBS/0.1% Tween-20, membranes were incubated in Odyssey Blocking Buffer diluted 1∶1 with PBS containing IRDye®800CW- or IRDye®680-conjugated secondary antibody (1 hour, 4°C). Bands were visualized by infrared imaging (Licor, Odissey) and images recorded as TIFF files for quantification with Adobe Photoshop software. Rabbit α-phospho-T202/Y204-ERK1/2 (Cell Signaling, # 9101); rabbit α-ERK1 (Santa Cruz, sc-93); rabbit polyclonal α-CSF-1R C-20 (Santa Cruz; sc-692); goat α-CSF-1 antibody N-16 (Santa Cruz, sc-1324); α-phospho-723-CSF-1R (Cell Signaling, # 3151); mouse α-vinculin (sigma; V9131); rabbit α-phospho-S63/73-c-Jun (Santa Cruz; sc16312); mouse monoclonal α-cyclin D1 (Santa Cruz, sc-8396); mouse monoclonal α-myc (Santa Cruz, sc-8396).

### Flow cytometry

Cells were detached by incubation in PBS containing 0.2% EDTA (pH 7.2), washed with PBS, pelleted and incubated in 20 µl of an anti-CSF-1R antibody (24A4, PE-conjugated; sc-02PE; Santa Cruz Biotechnology) [48] or isotype control antibody (IgG2b, sc-2873, Santa Cruz Biotechnology) for 45 minutes in the dark. After two washes with PBS followed by a 5 minutes centrifugation, cells were resuspended in 500 µl PBS and analyzed with a FACSCanto (Becton Dickinson). The percentage of positive cells was calculated by subtracting values obtained with isotype control antibody from those obtained with anti-CSF-1R antibody.

### Enzyme-Linked ImmunoSorbent Assay

CSF-1 was measured by ELISA (RayBio ELISA KIT Human MCSF), according to the manufacturer's instructions. The manufacturer claims that the minimum detectable dose of CSF-1 is typically less than 5 pg/ml (sensitivity of the methods). Supernatants were collected at cell confluency from cultures in complete medium. Each sample (100 µl) was assessed in duplicate.

### CSF-1R silencing with siRNA and measurement of DNA synthesis by [3H]thymidine uptake

Silencing was performed as previously described [40] with 100 nM SMART-pool siRNA for CSF-1R (NM_005211 mRNA, Dharmacon, cat. No M-003109-03), 100 nM SMART-pool siRNA for CSF-1 (NM_000757, Dharmacon, cat. No M-017514-00) or 100 nM siCONTROL non-targeting pool (Dharmacon, cat. n. D-001206-13) following the manufacturer's instructions. Transfection efficiency was 90%, as assessed by cotransfection with Cy3-labelled siGLO RISC-free siRNA (Dharmacon, cat. n. D-001600-01). One day after transfection, cells were serum starved for 24 hours before treatment with CSF-1 for 24 h. [3H]thymidine uptake analysis was performed as previously described [40].

### Measurement of DNA synthesis by bromodeoxyuridine uptake and immunofluorescence

Cells were seeded onto glass coverslips in complete medium for 24 hours and then incubated in the absence of FBS for 24 hours before being incubated in DMEM with or without 25 ng/ml CSF-1 or a 1∶50 dilution of a goat α-CSF-1 blocking or pre-immune serum [49] for further 24 hours. During the last 4 hours of incubation, bromodeoxyuridine (BrdU) was added to the culture (final concentration 10 nM) and then BrdU uptake was stopped by incubating cells in 4% formaldehyde/PBS for 10 minutes at RT. After washing in PBS, cells were incubated for 20 minutes with 2M HCl and then 0.1 M Na_2_B_4_O_7_ was added. Cells were washed with PBS and permeabilized by a 5 minute incubation in PBS containing 0.2% Triton X-100. After three washes in PBS cells were incubated with 10% horse serum in PBS/1%BSA for 45 minutes and then washed in PBS and incubated overnight at 4°C in a 1∶250 dilution of a mouse monoclonal α-BrdU antibody (Millipore, MAB3222) in PBS/1%BSA, washed with PBS and incubated with a 1∶800 dilution of an α-mouse Cy3-labelled secondary antibodies (Chemicon, AP192C). Cells were washed in PBS and incubated with 5 µg/ml Hoechst 33258 (Sigma) nuclear dye in PBS for 10 minutes. Following two washes in PBS, coverslips were mounted with propyl-thiogallate on glass slides and cells observed with a Leica DC200 microscope. Pictures were taken from 6 different field/sample (>600 cells were scored for each treatment) and the percentage of cells undergoing DNA synthesis was calculated by the ratio of the number of BrdU-positive cells to the total number of cells determined by Hoechst 33258 staining. Incubation with secondary antibody alone did not produce any significant fluorescence.

### Measurement of cell number by Crystal Violet staining

Cells were seeded in 12 multi-well plates and incubated for 24 hours in complete medium before serum starvation for further 24 hours. Cells were then treated in serum-free medium with imatinib (Gleevec, Glivec, Novartis, Basel, Switzerland) for 45 minutes and then with CSF-1 for further 48 hours. Cell were then washed twice with PBS and incubated for 10 minutes at RT with a Crystal Violet solution (0.5% Crystal violet, SIGMA, 30% ethanol and 3% formaldehyde). After extensive water washing, plates were allowed to dry and dye extracted by incubating with 1% SDS. Densitometric measurement was then performed at 550 nm.

### Quantitative Real-Time PCR (Q-PCR)

After total RNA extraction by TRIzol (Invitrogen) as specified by the manufacturer, 1 µg of total RNA/sample was submitted to reverse transcription with SuperScriptVILO-Reverse Transcriptase (Invitrogen) for 10 minutes at 25°C, 1 hour at 42°C and 5 minutes at 85°C utilizing 50 pmol random hexameric primers. The primers used were as follows: CSF-1R, C-term, for: 5′-CCTCGCTTCCAAGAATTGCA-3′, rev: 5′-CCCAATCTTGGCCACATGA-3′ (amplicon size 60 bp, designed to span the intron between exons 16 and 17); CSF-1R, N-term, for: 5′-GGAGGCTGCCCAGATCGT-3′ rev: 5′-GCGAGCTTGGTGTTGTTGTG-3′ (amplicon size 60 bp, designed to span the intron between exons 4 and 5); cyclin D1 (*CCND1*), for: 5′-GAGAGGAAGCGTGTGAGGCGGTAG-3′, rev: 5′-GGATGCTGGAGGTGCGAGGA-3′; cyclin G1 (*CCNG1*), for: 5′-CCAGCTGAATGCCCTGTTG-3′, rev: 5′-AGTCTCAAACCACAGACCTTTGG-3′; cyclin H (*CCNH*), for: 5′-GAGGAGCAGCTGGCAAGACT-3′, rev: 5′-ACGGCTTTGCATCTGAATTTG-3′; cyclin I (*CCNI*), for: 5′-CATCTCAACATTTGGCAGTCCTT-3′, rev: 5′-GAAGTTGGTTGCAGGCCATAC-3′; rRNA 18S, for: 5′-CGGCTACCACATCCAAGGAA-3′, rev: 5′-GCTGGAATTACCGCGGCT-3′ (amplicon size 180 bp); GAPDH, for: 5′-AACAGCC TCAAGATCATCAGCAA-3′, rev: 5′-CAGTCTGGGTGGCAGTGAT-3′. CSF-1R mRNA expression was assessed by Q-PCR (2 minutes 50°C, 5 minutes 95°C, 40 cycles at 95° C for 15 seconds and 60°C for 1 minute) with the ABI Prism 7500 Sequence Detection System (Applied Biosystem) using Power SYBR® Green PCR master mix (Applied Biosystem). A melting curve analysis was performed to discriminate between specific and non-specific PCR products. Alternatively, amplified product size was verified by running in 3–4% agarose gels. The housekeeping 18S rRNA and/or GAPDH genes were used as internal references for normalization. The relative expression of CSF-1R, with respect to SKBR3 cells chosen as calibrator, was calculated by using a comparative threshold cycle method and the formula 2^(−ΔΔCt)^ [50].

### Analysis of StellARray Gene Expression

SKBR3 cells were transfected with the indicated siRNA and cultured in complete medium for 72 hours. Cells were then lysed and total RNA extracted. Gene expression analysis was performed with Q-PCR Arrays (Human Cell Cycle Tox and Cancer 96 StellARray™ qPCR array 00188263 Fast 96 Well, Lonza). A list of these 96 genes is available online at http://array.lonza.com/plate/00188263/. The housekeeping rRNA 18S gene was used as internal reference for normalization. Fold change was calculated as described above. Data reported (+SEM) were obtained from four independent experiments.

## Results

**Figure 1:**
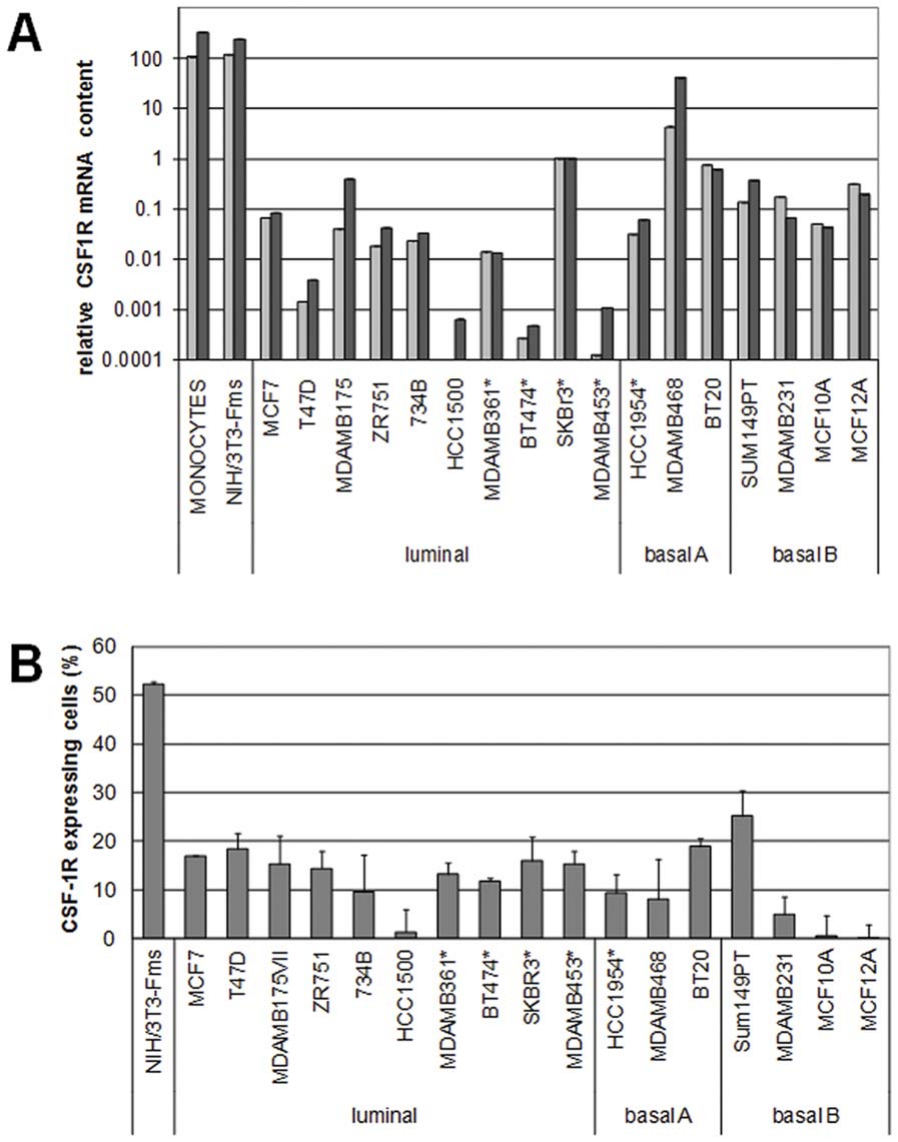
Expression of CSF-1R in breast cancer cell lines. (A) Routinely cultured cells were lysed and total RNA extracted. Q-PCR was performed using two different sets of primers (C-terminus, light gray; N-terminus, dark grey). The 18S rRNA was used for normalization and SKBR3 were chosen as calibrator. Data represent the mean (± SD) of three independent experiments. Asterisks indicate the HER2-overexpressing cell lines. (B) Routinely cultured cells were processed and subjected to flow cytometry with a rat monoclonal anti-CSF-1R antibody. Columns represent the percentage of CSF-1R-positive cells within the bulk population analyzed (gated in order to exclude debris and cellular aggregates). Data represent the mean (± SEM) of 3 independent experiments. Asterisks indicate the HER2-overexpressing cell lines. One-way ANOVA among different subtypes showed no differences.

The expression of CSF-1R and/or CSF-1 in human breast carcinomas has been documented in both cell lines and tumors samples [8]–[10], [12], [15]. However, whether their expression is restricted to one molecular subtype has not been studied. To address this issue, we performed Q-PCR for CSF-1R mRNA on 17 cell lines endowed with different molecular profiles and referred to as luminal, basal A and basal B subtypes (Figure 1A) [41], [42], [51], [52]. These experiments indicated that *CSF1R* is expressed, although at different levels, in all cell lines tested.

The presence of cell surface CSF-1R protein was then verified by flow cytometry (Figure 1B, S1A). All the cell lines tested, except HCC1500, MCF10A and MCF12A, expressed appreciable cell surface CSF-1R. However, MCF10A and MCF12A cells expressed high levels of CSF-1R mRNA. We hypothesized that these differences are due to ligand-induced down-regulation of the receptor (see below). Furthermore, ectopic CSF-1 activates ERK1/2 in these cells pointing to the presence of functional CSF-1R. In contrast, HCC1500 cells express very low CSF-1R mRNA levels and did not respond to CSF-1 as monitored by activation of ERK1/2 (Figure S3), PI3K or ERK5 (not shown). HCC1500 is, therefore, the only breast cancer cell line of the panel analyzed that we consider CSF-1R negative. The analysis of mean fluorescence intensity produced similar results (not shown). No significant differences in cell surface CSF-1R expression among subtypes were found by one-way ANOVA (not shown).

The cell lines were also tested for CSF-1 expression by measurement of secreted CSF-1 (by ELISA and western blotting) and of CSF-1 in cell lysates (by western blotting). CSF-1 was detected in all the cell lines tested (Table 1). It is of note that we were unable to detect CSF-1 in SKBR3 supernatants by ELISA, a finding that is at variance with previous reports [53]. The detection of CSF-1 in total cell lysates of SKBR3 indicates that these cells, which are known to express CSF-1 [15], may express membrane-bound CSF-1.

**Table 1:** Expression and production of CSF-1 in breast cancer cells.

	luminal										basal A			basal B				
	MCF7	T47D	MDAMB175	ZR751	734B	HCC1500	MDAMB361*	BT474*	SKBR3*	MDAMB453*	HCC1954*	MDAMB468	BT20	Sum149PT	MDAMB231	MCF10A	MCF12A	NIH/3T3-Fms
ELISA	−	−	−	−	−	−	−	−	−	−	+	−	−	+	++	++	nd	(−)
WB	+	+	+	+	+	+	+	+	+	+	++	+	+	++	++	+++	+++	+++

ELISA of cell culture supernatants with an antibody directed to biologically-active human CSF-1; −: below assay sensitivity; +: <50 pg/ml; ++: >50 pg/ml; nd: not determined. WB: western blotting of cell lysates and supernatants using a goat polyclonal antibody (N-16). +, ++, +++ refer to the presence of detectable, marked or large amounts of CSF-1-specific bands, respectively.

**Figure 2:**
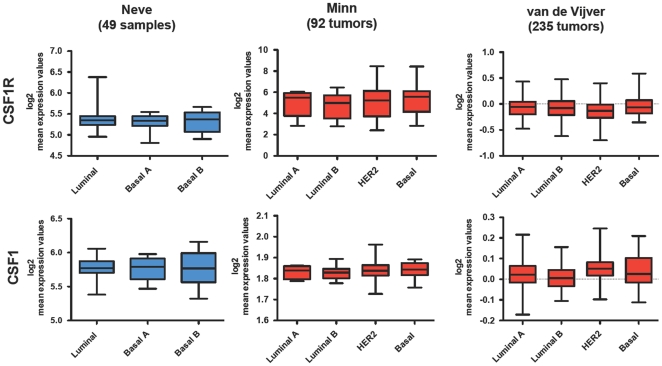
*In silico* analysis of CSF1 and CSF1R genes expression in breast cancer datasets. CSF1 and CSF1R transcripts levels are shown for breast cancer cell lines dataset (blue) from Neve et al. [41] and for two independent breast tumor datasets (red) from Minn et al. [43] and van de Vijver et al. [44]. One-way ANOVA and the Bonferroni test did not show any statistical significant difference among the subtypes within the dataset.

The data reported in Figure 1 and Table 1 indicated that all the cell lines tested express CSF-1 and that 16/17 express CSF-1R. In addition, we performed *in silico* analysis on publicly available gene expression profiling datasets (Figure 2) [41], [43], [44]. This analysis indicated that the mean expression of *CSF1* and *CSF1R* genes did not vary significantly (assessed by One-way ANOVA analysis) among breast cancer subtypes in either cell lines or in tumors samples. Moreover, *in silico* analysis of comparative genomic hybridization datasets relative to 49 breast cancer cell lines [41] and 67 primary tumors [47] indicated the absence of CSF-1R or CSF-1 gene amplifications (Figure S2).

**Figure 3:**
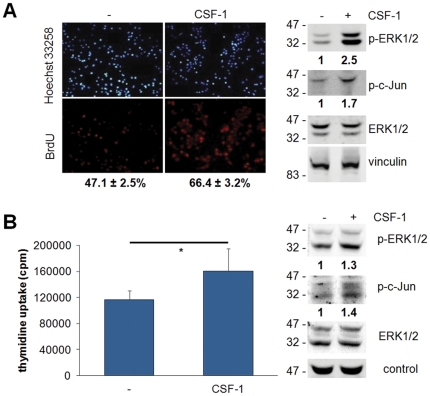
Effects of CSF-1 on the proliferation of breast cancer cells. MDAMB468 (A) or SKBR3 (B) cells were cultured in DMEM without serum for 24 hours and then for additional 24 hours with DMEM with or without 25 ng/ml CSF-1. Cells were scored for (A) BrdU or (B) tritiated thymidine uptake or lysed and protein subjected to immunoblotting with the indicated antibodies. (A) Values reported under the pictures are percentages (± SEM) of BrdU-positive nuclei, normalized to total nuclei labeled by Hoechst 33258, from 5 independent experiments; **, Student's *t* test: p<0.01. (B) Data represent mean (± SEM) of one out of 3 representative experiments; *, Student's *t* test: p<0.05. Densitometric values of bands (normalized for loading control) are reported as ratios between the CSF-1-treated and untreated value, set as 1.

To determine whether CSF-1R transduces proliferative signals in breast cancer cells we chose two cell lines from different subtypes (Figure 3). When basal MDAMB468 cells were exposed to exogenous CSF-1, their proliferation increased by 40% and ERK1/2 and c-Jun phosphorylation were markedly increased. In addition CSF-1 administration increased cell proliferation and ERK1/2 and c-Jun phosphorylation of luminal SKBR3 cells. It is of note that this cell line is characterized by a high basal ERK1/2 phosphorylation suggesting an autocrine CSF-1/CSF-1R loop and/or activation of CSF-1R-independent signaling pathways. The relevance of ERK1/2 in CSF-1R signaling was highlighted by the fact that enhancement of ERK1/2 phosphorylation upon CSF-1 treatment was found in 10 out of 17 breast cancer cell lines (Figure S2).

**Figure 4:**
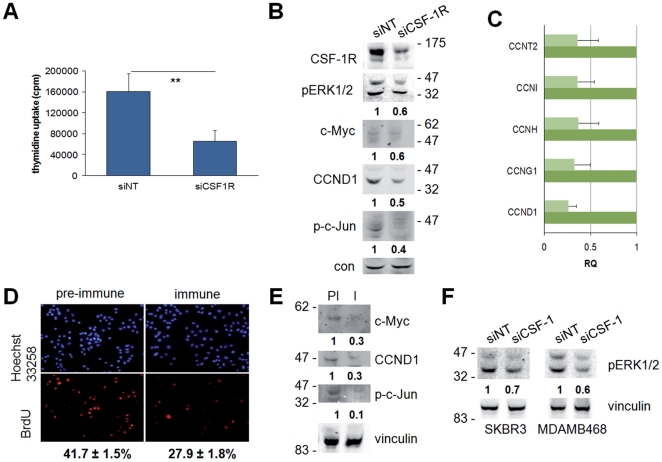
Effects of CSF-1/CSF-1R inhibition on the proliferation and ERK1/2 phosphorylation of breast cancer cells. (A–C, F) SKBR3 or (F) MDAMB468 cells were transfected with the indicated siRNA and incubated for 24 hours. (A) Cells were then serum-starved for a further 24 hours and treated with 25 ng/ml CSF-1 for 24 hours, and tritiated thymidine uptake measured. Data represent mean (± SEM) of one out of 3 representative experiments; **, Student's *t* test: p<0.01. (B, C, F) 72 hours post transfection cells were lysed and total protein or RNA extracted. (B, F) Protein lysates were subjected to immunoblotting with the indicated antibodies. Densitometric values of bands (normalized for loading control) are reported as ratios between the siCSF-1R and the siNT value, set as 1. (C) Q-PCR was performed for the indicated genes. Data were normalized against GAPDH. siNT-treated samples (dark green) were chosen as calibrator over the siCSF1R-treated samples (light green). Data represent the mean (± SEM) from three independent experiments performed in triplicates. (D, E) SKBR3 cells were cultured in DMEM without serum for 24 hours and then for 24 hours with 25 ng/ml CSF-1, pre-incubated for 1 hour with a 1∶50 dilution of CSF-1-blocking anti-serum (I) or control pre-immune serum (PI). Cells were then scored for BrdU uptake or lysed. (D) Values reported under the pictures, from 5 independent experiments, are percentages (± SEM) of BrdU-positive nuclei, normalized to total nuclei labeled by Hoechst 33258; ***, Student's *t* test: p = 0.0004. (E) Protein lysates were subjected to immunoblotting with the indicated antibodies. Densitometric values of bands (normalized for loading control) are reported as ratios between the I and the PI value, set as 1.

To further characterize CSF-1R involvement in breast cancer cell proliferation we treated breast cancer cells (Figure 4) with CSF-1R siRNA (Figure S1A and B). CSF-1R silencing in SKBR3 cells markedly impaired cell proliferation in the presence of ectopic CSF-1 (Figure 4A), reduced the phosphorylation of ERK1/2 and c-Jun as well as the expression of c-Myc, cyclin D1 (Figure 4B) and several other cyclins (Figure 4C and Table 2).

**Table 2:** Effect of CSF-1R silencing on the expression of cyclin genes.

ID	Gene name	fold change	p (*t* Student's)
**890**	cyclin A2	0.438248	**0.042839**
**891**	cyclin B1	0.30756	**0.030438**
**595**	cyclin D1	0.488026	0.073304
**894**	cyclin D2	undetectable	
**896**	cyclin D3	0.378628	**0.047503**
**898**	cyclin E1	0.352627	0.068412
**9134**	cyclin E2	0.387063	0.06905
**900**	cyclinE2	0.44102	**0.02955134**
**901**	cyclin G1	0.517056	**0.034455**
**902**	cyclin G2	11.70318	0.196287
**10983**	cyclin H	0.364232	**0.009274**
**8812**	cyclin I	0.268787	**0.00119**
**905**	cyclin K	0.333527	**0.044601**
**890**	cyclin T2	0.30201	**0.00467**

To test whether an autocrine CSF-1/CSF-1R loop exists in breast cancer cells (Figure 4D) we used a CSF-1-blocking antiserum (Figure S1C) [49]. SKBR3 cells treated for 24 hours with the CSF-1-blocking antiserum showed a 35% reduction in proliferation when compared to the cells treated with the control serum (pre-immune) (Figure 4D). In the same experiments, the CSF-1-blocking antiserum markedly inhibited c-Jun phosphorylation and reduced cyclin D1 and c-Myc expression (Figure 4E). This autocrine signaling sustained ERK1/2 activation in both SKBR3 and MDAMB468 cells (Figure 4F) as demonstrated by the fact that CSF-1 silencing partially impaired basal ERK1/2 phosphorylation. CSF-1 silencing was confirmed by Q-PCR (not shown). The results shown in Figure 3 and 4 indicated the involvement of ERK1/2 and its downstream targets (i.e. c-Jun, cyclin D1 and c-Myc) in CSF-1-induced cell proliferation in breast cancer cells.

**Figure 5:**
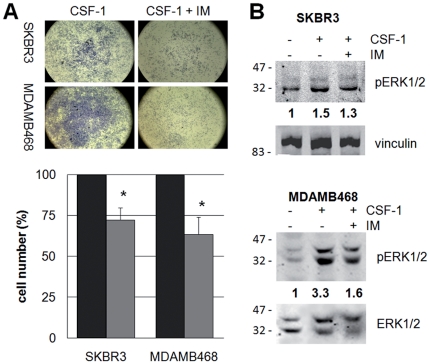
Effects of imatinib on breast cancer cell survival and ERK1/2 phosphorylation in response to CSF-1. 24 hours serum deprived SKBR3 or MDAMB468 cells were treated for 45 minutes with (light gray columns, right pictures) or without (dark gray columns, left pictures) 10 µM imatinib (IM) and then with 25 ng/ml CSF-1 for (A) 48 hours or (B) 10 minutes. (A) Cells were scored for cell viability by crystal violet staining. Data represent percentages (± SEM) of crystal violet staining normalized for IM-untreated cells from 4 independent experiments; *, Student's *t* test: p<0.05. (B) Cells were lysed and protein lysates subjected to immunoblotting with the indicated antibodies. Densitometric values of bands (normalized for loading control) are reported as ratios between treated and untreated value, set as 1.

The fact that CSF-1 promotes proliferation in breast cancer cells prompted us to determine whether their growth is sensitive to tyrosine kinase inhibitors used in the clinic. Imatinib (IM) is known to inhibit CSF-1R activity [34]. We found, in keeping with previous data [34], that 10 µM IM was necessary to prevent CSF-1-induced CSF-1R phosphorylation in NIH/3T3-Fms cells (Figure S1D). When 10 µM IM was given to SKBR3 and MDAMB468 breast cancer cell lines for 48 hours in the presence of CSF-1, cell number was markedly reduced, as determined by crystal violet staining (Figure 5A). Further, IM was able to prevent in part the activation of ERK1/2 induced by CSF-1 confirming the involvement of ERK1/2 in the proliferative effect of CSF-1 in breast cancer cells (Figure 5B). IM treatment alone did not decrease ERK1/2 phosphorylation (not shown).

## Discussion

Although the expression of CSF-1/CSF-1R has been previously documented in breast cancer and shown to correlate with poor prognosis, few studies have been performed to understand the role of CSF-1R-dependent signaling in the proliferation of breast cancer cells or other solid tumors [24], [54], [55]. In the present study we found that: i) breast cancer cell lines consistently express CSF-1 and CSF-1R; ii) the CSF-1/CSF-1R pair sustains the proliferation of breast cancer cell lines; iii) ERK1/2 is downstream CSF-1R in proliferating breast cancer cells.

CSF-1R sustains breast cancer cells proliferation, as highlighted in two cell lines of different molecular subtypes. Indeed, interfering with CSF-1/CSF-1R signaling, either by targeting the receptor or by blocking the ligand binding, impacts on breast cancer cell proliferation. This proliferation was induced either by ectopic CSF-1, that mimic CSF-1 produced by fibroblasts and monocytes/macrophages associated with the tumor, or autocrine CSF-1. We found that CSF-1R activation sustained the expression of cyclin D1 and c-Myc and activated c-Jun, which are established CSF-1 downstream targets in other cell types [56]. Interestingly, the expression of several cyclins was decreased following CSF-1R silencing in SKBR3 cells, indicating that in these cells CSF-1R-dependent signaling sustains the progression across different phases of the cell cycle.

We also found that CSF-1R-induced proliferation involves ERK1/2 activation in SKBR3 and MDAMB468 cells. Our data are in line with previous studies in which breast cancer cell lines expressing ectopic CSF-1R showed increased expression of cyclin D1 as a consequence of ERK1/2 activation upon CSF-1 administration [57]. Our results therefore support the existence of a proliferative pathway elicited by CSF-1/CSF-1R that acts through ERK1/2 thereby inducing c-Jun activation as well as c-myc and cyclin D1 expression. We also found that ERK5 [40], [58] is activated in a restricted number of cell lines following CSF-1 treatment (not shown). PI3K [59] was not activated by CSF-1 in any of the cell lines tested. In this respect, it is to note, however, that both ERK5 and PI3K pathways are often constitutively active in breast cancer cells [58], [60], possibly masking a response to CSF-1.

Our data show that 16 cell lines among 17 tested express CSF-1 and CSF-1R, although at different levels. Moreover, gene expression profiling datasets show that *CSF1* and *CSF1R* expression is a general feature of breast cancer cells. These findings support the possibility to target CSF-1R signaling in a large proportion of breast cancers, independently of their molecular subtype. This is particularly relevant for tumors that are classify as of the basal-like subtype. Triple-negative (ER-, PR-, HER2-negative) breast cancers make up the majority of this subgroup [41], [61] and are generally unresponsive to standard treatments, i.e. tamoxifen, aromatase inhibitors and herceptin. Although triple-negative breast cancers can be treated with chemotherapy, early relapse and metastasis is common and therefore the need of potential targets for this tumors is of high priority.

The data obtained from our experiments and *in silico* analysis indicate that CSF-1R is not overexpressed (when compared to human monocytes) nor amplified [62] and therefore support previous reports [6] that the oncogenic potential of CSF-1R is due to its co-expression with CSF-1. Furthermore, this conclusion is further established by the fact that CSF-1R-dependent proliferation of SKBR3 cells is impaired when the CSF-1/CSF-1R interaction is prevented. ERK1/2 is apparently involved in autocrine-induced proliferation as silencing CSF-1 or CSF-1R decreased ERK1/2 activation in both SKBR3 and MDAMB468. Moreover, ERK1/2 was constitutively active in several cell lines where CSF-1 exposure had a minor effect on the basal ERK1/2 phosphorylation. The low level or absent responsiveness to CSF-1 in these cell lines may be the consequence of autocrine CSF-1 production (as demonstrated for SKBR3) and/or of activation of CSF-1R-independent signaling pathways.

IM is a tyrosine kinase inhibitor in use for the clinical management of chronic myeloid leukemia and has been shown to inhibit several different kinases, including CSF-1R. We found that IM impairs the proliferation of breast cancer cell lines in the presence of CSF-1. As IM targets kinases other than CSF-1R our experiments do not prove that the effects shown are only due to CSF-1R inhibition. However, they support the concept that targeting CSF-1R with tyrosine kinase inhibitors can effectively block CSF-1-dependent growth stimulation. This is particularly relevant when considering strategies to interfere with autocrine CSF-1-dependent proliferation.

In conclusion, the evidence we provided for the expression and functional role of the CSF-1/CSF-1R pair in breast cancer, together with the established role of CSF-1/CSF-1R in breast cancer motility and invasiveness [29]–[31], indicate that CSF-1R targeting may be pursued therapeutically, irrespective of breast cancer subtype, at either early or late stages of tumor progression.

## Supporting Information

Figure S1 **Settlement of CSF-1 or CSF-1R targeting using NIH/3T3 cells expressing ectopic CSF-1R.** (A) NIH/3T3-Fms cells were transfected with the indicated siRNA. Total protein lysates obtained at the indicated times were subjected to immunoblotting with the indicated antibodies. Densitometric values of bands (normalized for loading control) are reported as ratios between the siCSF1R and the siNT value, set as 1. 72 hours post-transfection cells were analyzed by flow cytometry. Percentages of CSF-1R-positive cells are reported. (B) NIH/3T3-Fms cells were transfected with the indicated siRNA and incubated for 24 hours. Cells were then serum-starved for further 24 hours and treated with (CSF-1) or without (−) 25 ng/ml CSF-1 for 24 hours, and tritiated thymidine uptake measured. Data represent mean (± SEM) of one of 3 representative experiments; ** and ***, Student's *t* test: p<0.01, p<0.001, respectively. (C) NIH/3T3-Fms cells were incubated with or without 25 ng/ml CSF-1 for 10 minutes. Before cell treatment, CSF-1 had been incubated for 1 hour at 37°C in the absence (−) or the presence of a 1∶50 dilution of a CSF-1-blocking anti-serum (I) or pre-immune serum (PI). Total protein lysates were subjected to immunoblotting with the indicated antibodies. Densitometric values of bands (normalized for loading control) are reported as ratios between the siCSF-1R and the correspondent siNT value, set as 1. (D) NIH/3T3-Fms cells were cultured for 24 hours without serum and then for 45 minutes with the indicated doses of imatinib (IM) before treatment with or without 25 ng/ml CSF-1 for 10 minutes. Cells were then lysed and protein subjected to immunoblotting with the indicated antibodies.
(TIF)

Figure S2 **CSF-1 and CSF-1R gene copy number in breast cancer cell lines and tumor samples.** Data have been collected from CGH experiments performed by others with breast cancer cell lines (left) [41] or tumor samples (right) [47]. Whisker graphs represent median, 25- and 75- percentile, min and max values.
(TIF)

Figure S3 **CSF-1R signaling induces ERK1/2 phosphorylation in breast cancer cell lines.** Serum-deprived cells (24 hours) were incubated with or without CSF-1 (25 ng/ml) for 10 minutes and lysed in RIPA buffer. Total protein lysates were subjected to immunoblotting with the indicated antibodies. N3F: NIH/3T3-Fms cells. Densitometric values of bands (normalized for loading control) are reported as ratios between the CSF-1-treated and the untreated value, set as 1. Threshold for activation was arbitrary set at ≥1.2.
(TIF)
