# Supplementary figures and images for: The Colony-Stimulating Factor-1 (CSF-1) Receptor Sustains ERK1/2 Activation and Proliferation in Breast Cancer Cell Lines
Source: PLoS One. 2011 Nov 9;6(11):e27450. doi: 10.1371/journal.pone.0027450 (PMC3212567; doi:10.1371/journal.pone.0027450)

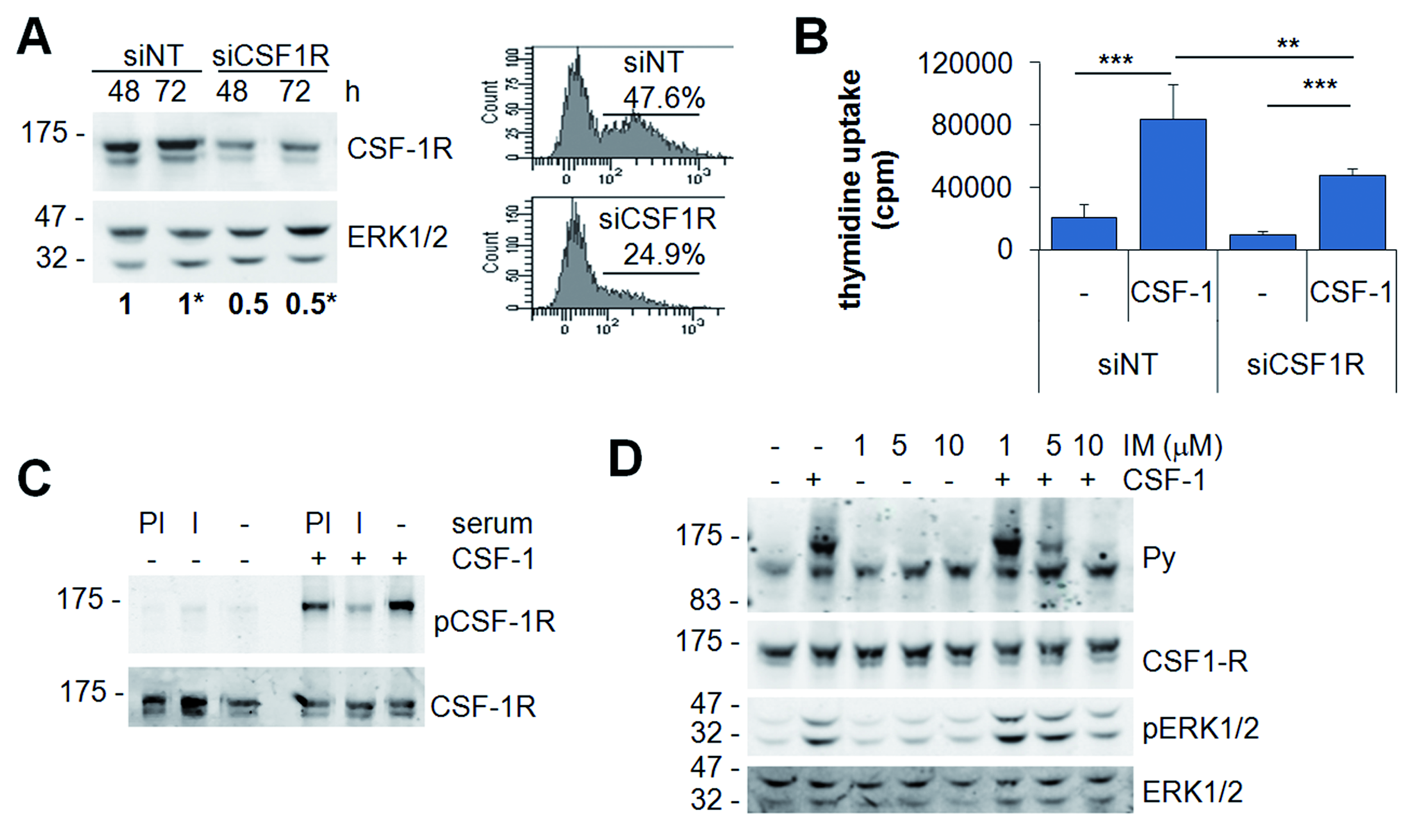

Supplement: Figure S1 — Settlement of CSF-1 or CSF-1R targeting using NIH/3T3 cells expressing ectopic CSF-1R. (A) NIH/3T3-Fms cells were transfected with the indicated siRNA. Total protein lysates obtained at the indicated times were subjected to immunoblotting with the indicated antibodies. Densitometric values of bands (normalized for loading control) are reported as ratios between the siCSF1R and the siNT value, set as 1. 72 hours post-transfection cells were analyzed by flow cytometry. Percentages of CSF-1R-positive cells are reported. (B) NIH/3T3-Fms cells were transfected with the indicated siRNA and incubated for 24 hours. Cells were then serum-starved for further 24 hours and treated with (CSF-1) or without (−) 25 ng/ml CSF-1 for 24 hours, and tritiated thymidine uptake measured. Data represent mean (± SEM) of one of 3 representative experiments; ** and ***, Student's t test: p<0.01, p<0.001, respectively. (C) NIH/3T3-Fms cells were incubated with or without 25 ng/ml CSF-1 for 10 minutes. Before cell treatment, CSF-1 had been incubated for 1 hour at 37°C in the absence (−) or the presence of a 1∶50 dilution of a CSF-1-blocking anti-serum (I) or pre-immune serum (PI). Total protein lysates were subjected to immunoblotting with the indicated antibodies. Densitometric values of bands (normalized for loading control) are reported as ratios between the siCSF-1R and the correspondent siNT value, set as 1. (D) NIH/3T3-Fms cells were cultured for 24 hours without serum and then for 45 minutes with the indicated doses of imatinib (IM) before treatment with or without 25 ng/ml CSF-1 for 10 minutes. Cells were then lysed and protein subjected to immunoblotting with the indicated antibodies. (TIF) [file pone.0027450.s001.tif]

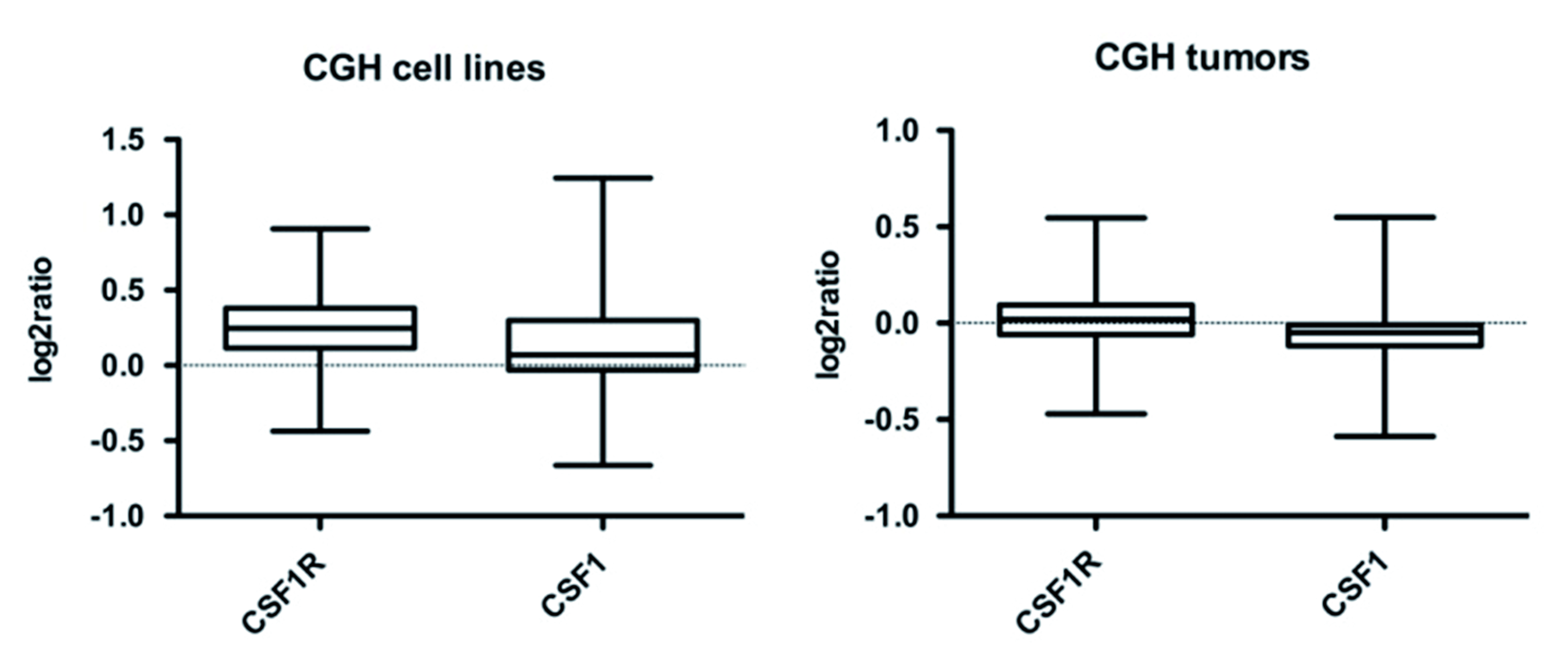

Supplement: Figure S2 — CSF-1 and CSF-1R gene copy number in breast cancer cell lines and tumor samples. Data have been collected from CGH experiments performed by others with breast cancer cell lines (left) [41] or tumor samples (right) [47]. Whisker graphs represent median, 25- and 75- percentile, min and max values. (TIF) [file pone.0027450.s002.tif]

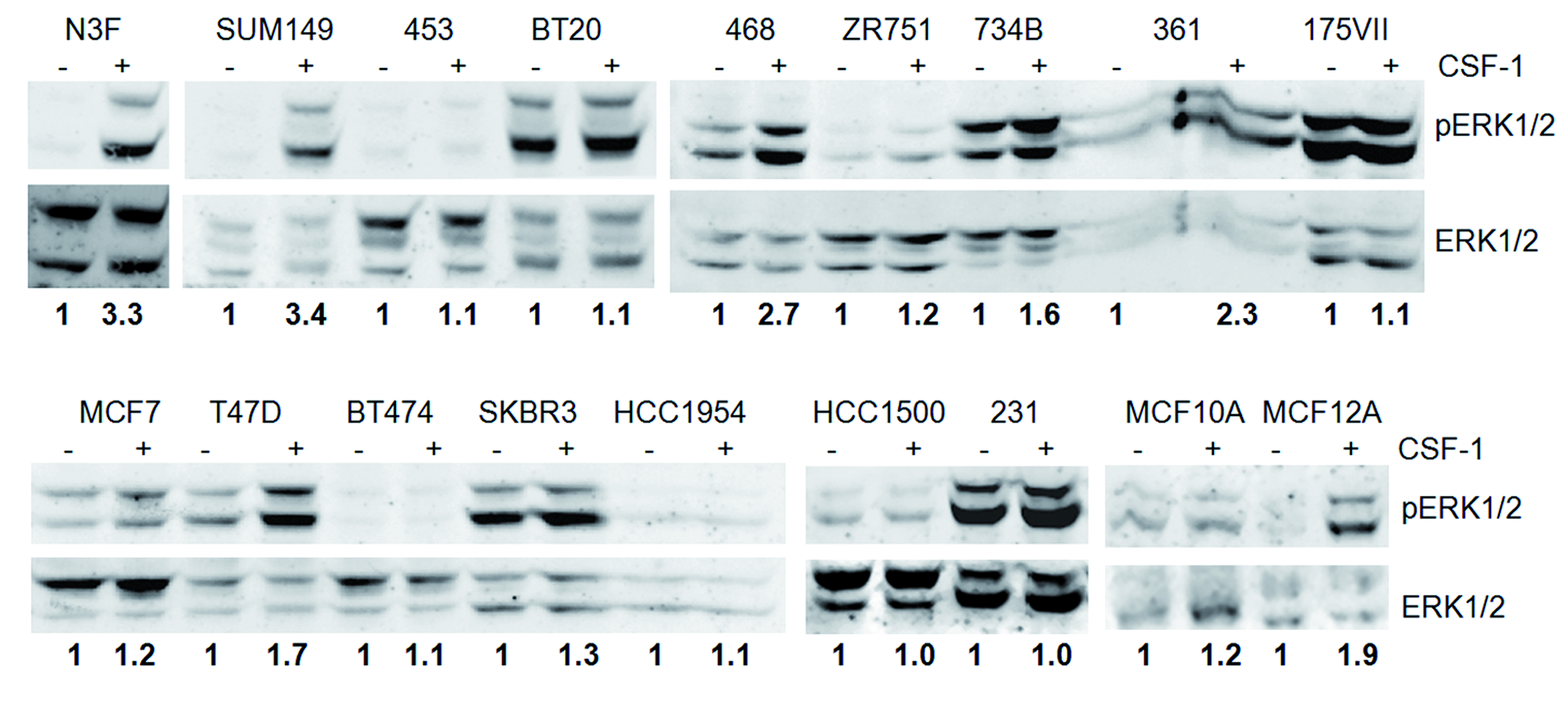

Supplement: Figure S3 — CSF-1R signaling induces ERK1/2 phosphorylation in breast cancer cell lines. Serum-deprived cells (24 hours) were incubated with or without CSF-1 (25 ng/ml) for 10 minutes and lysed in RIPA buffer. Total protein lysates were subjected to immunoblotting with the indicated antibodies. N3F: NIH/3T3-Fms cells. Densitometric values of bands (normalized for loading control) are reported as ratios between the CSF-1-treated and the untreated value, set as 1. Threshold for activation was arbitrary set at ≥1.2. (TIF) [file pone.0027450.s003.tif]
